# Impact of respiratory support on time to achieve full oral feeding in very low birth weight infants: a retrospective study

**DOI:** 10.3389/fped.2026.1937237

**Published:** 2026-09-29

**Authors:** Qi Xu, Xiaotong Zhang, Jiajing Chi, Minmin Li

**Affiliations:** 1 Department of Neonatology, Shandong Provincial Hospital Affiliated to Shandong First Medical University, Jinan, China; 2 The School of Nursing, Shandong First Medical University, Jinan, China; 3 Department of Cardiovascular Surgery, Shandong Provincial Hospital Affiliated to Shandong First Medical University, Jinan, China

**Keywords:** full oral feeding, non-invasive ventilation, propensity score matching, respiratory support, very low birth weight infant

## Abstract

**Background:**

Very low birth weight infants often require respiratory support, which may delay full oral feeding. The independent roles of invasive versus non-invasive support are unclear. This study used propensity score matching to assess their associations with feeding time.

**Objective:**

To investigate the impact of different durations of respiratory support on the time to achieve full oral feeding in very low birth weight infants, and to provide evidence for early identification of high-risk infants and optimization of respiratory support management.

**Methods:**

This retrospective cohort study included 753 VLBWI admitted to a tertiary hospital from 2018 to 2024. Infants were dichotomized into long- and short-FOF groups according to the median time to FOF. Propensity score matching (1:1 nearest-neighbor, caliper = 0.1) balanced baseline confounders, yielding 179 matched pairs. Durations of initial invasive mechanical ventilation, non-invasive positive pressure ventilation, and total positive pressure support were compared. Multivariable conditional logistic regression was applied to the matched data to identify independent risk factors for prolonged FOF.

**Results:**

After PSM, the long-FOF group exhibited significantly longer NIPPV duration (median: 19 vs. 13 days, *P* < 0.001) and total support duration (23 vs. 15 days, *P* < 0.001) compared to the short-FOF group, while initial IMV duration showed no significant difference (*P* = 0.224). Multivariable analysis demonstrated that both NIPPV duration (aOR = 1.035, 95% CI: 1.015-1.055) and total support duration (aOR = 1.033, 95% CI: 1.015-1.052) were independent risk factors for delayed FOF. In contrast, the independent association of initial IMV duration was model-dependent and not consistently significant after matching.

**Conclusion:**

The duration of non-invasive positive pressure respiratory support is significantly associated with delayed achievement of full oral feeding in very low birth weight infants.

## 1 Introduction

The World Health Organization (WHO) reports that approximately 13.4 million preterm infants are born globally each year (1). Due to their immaturity, these infants often require treatment and care in the neonatal intensive care unit (NICU). Preterm infants are susceptible to respiratory diseases such as neonatal respiratory distress syndrome and bronchopulmonary dysplasia because of their underdeveloped respiratory systems, which can lead to respiratory failure (2). Consequently, they frequently require artificial airway establishment with mechanical ventilation and are initially fed via tube feeding (3). The achievement of full oral feeding (FOF) capability is one of the critical criteria for hospital discharge in preterm infants (4). Swallowing function begins to develop at around 16 weeks of gestation, and healthy full-term newborns can typically meet their nutritional needs through sucking immediately after birth (5). However, preterm infants, especially very low birth weight infants (VLBWI) with a birth weight <1500 g, face challenges such as feeding intolerance and poor autonomous sucking ability due to immature gastrointestinal function and oropharyngeal coordination (6). These feeding difficulties can not only lead to growth retardation during the neonatal period but are also associated with long-term neurodevelopmental outcomes and metabolic health risks (7). Furthermore, delays in feeding progression directly result in prolonged hospital stays, increased medical costs, and greater family burdens (4). Wang et al. (8) found in their chart review that 32.2% of infants born at 35–36 weeks of gestation experienced feeding difficulties, and 76% of those had extended hospital stays as a result.

Multiple factors influencing the progression to oral feeding in preterm infants have been identified, including gestational age, birth weight, complications such as bronchopulmonary dysplasia, patent ductus arteriosus, necrotizing enterocolitis, as well as non-medical factors like socioeconomic status and parental involvement (9, 10). Among these, respiratory diseases and the required respiratory support are widely recognized as significant risk factors for delayed oral feeding (9). A meta-analysis (11) indicated that the duration of positive pressure respiratory support is a major determinant of the time to achieve FOF in preterm infants. However, existing studies often treat respiratory support as a single or composite variable, failing to adequately distinguish the independent effects and potential interactions between invasive and non-invasive respiratory support on feeding progression. Moreover, due to considerable sample heterogeneity and insufficient control for baseline confounders in most studies, the robustness of these conclusions and the reliability of causal inference require further validation.

To more precisely evaluate the impact of respiratory support on oral feeding progression in preterm infants, this single-center study utilized propensity score matching to balance baseline characteristics between groups with different feeding outcomes. We aimed to separately and jointly examine the effects of the duration of initial invasive mechanical ventilation, non-invasive positive pressure support, and total positive pressure support on feeding outcomes. Through comparative analysis before and after matching, this study seeks to reduce confounding bias, enhance the reliability of causal inference, and provide an evidence-based foundation for the early identification of high-risk infants and the optimization of respiratory support strategies.

## 2 Materials and methods

### 2.1 Study design

This was a single-center retrospective cohort study. Data were extracted from the online database of the Chinese Northern Neonatal Network (SNN) (http://www.snn-med.com). The study population comprised VLBWIs admitted to the NICU of a tertiary hospital between 2018 and 2024. Inclusion criteria were: (1) birth weight < 1,500 g; (2) born in the study hospital or transferred from another hospital within 24 h after birth. Exclusion criteria were: (1) presence of congenital gastrointestinal malformations or genetic metabolic diseases; (2) death within the first week of life, occurrence of spontaneous intestinal perforation, or withdrawal of life-sustaining treatment due to non-medical family decisions; (3) incomplete clinical data.

### 2.2 Population enrollment and ethical considerations

This was a single-center retrospective cohort study. The study population comprised very low birth weight infants admitted to the NICU of a tertiary hospital between 2018 and 2024. Written informed consent was obtained from the guardians of all enrolled infants. The study protocol was reviewed and approved by the hospital's Ethics Committee (Approval No. LCYJ: 2019132)

### 2.3 Data collection

Clinical data during hospitalization were collected via the SNN online platform and medical records. General information included: gestational age, birth weight, sex, mode of delivery, plurality, small for gestational age (SGA) status, and 5-minute Apgar score. Perinatal data included: maternal age, maternal hypertensive disorders of pregnancy, gestational diabetes mellitus, gestational hypothyroidism, and antenatal corticosteroid use. Respiratory-related factors included: whether initial invasive mechanical ventilation (IMV) was used, whether non-invasive positive pressure ventilation (NIPPV) support was used, duration of initial IMV, duration of NIPPV support, and total duration of positive pressure respiratory support.

### 2.4 Study variable definitions

1. Time to Achieve Full Oral Feeding (FOF): Defined as the postnatal day when all milk volumes were taken orally (or the feeding tube was removed) and adequate weight gain was sustained over 24 h (10). Infants were divided into a “long-FOF group” and a “short-FOF group” based on the median time to achieve FOF. Infants with a time ≥ the median were assigned to the long-FOF group, and the others to the short-FOF group.
2. Initial Invasive Mechanical Ventilation: This is a binary variable referring to whether the initial mode of mechanical ventilation received after birth was invasive (either invasive high-frequency mechanical ventilation or invasive conventional mechanical ventilation). This definition aimed to capture the starting point of respiratory support strategy. In clinical practice, the vast majority of VLBWIs requiring respiratory support undergo sequential therapy from invasive to non-invasive modes as their condition evolves (12). Therefore, cases receiving only invasive ventilation throughout the entire hospitalization are extremely rare. To more accurately reflect the initial disease severity and treatment decisions, and to avoid confounding due to subsequent mode transitions, this study selected the initial ventilation mode as the key exposure variable, rather than the use of “invasive mechanical ventilation” over the entire hospitalization period. This approach aids in a clearer analysis of the impact of interventions during the initial respiratory support phase on the maturation of later feeding function.
3. Non-invasive Mechanical Ventilation: Included the following modalities: non-invasive high-frequency positive pressure ventilation (HFPPV), non-invasive positive pressure ventilation (NIPPV), bi-level positive airway pressure (BiPAP), continuous positive airway pressure (CPAP), and nasal high-flow oxygen therapy (HFNC).
4. Total Duration of Positive Pressure Respiratory Support: The sum of the duration of invasive mechanical ventilation and non-invasive positive pressure respiratory support during hospitalization.

### 2.5 Statistical analysis

Data were analyzed using IBM SPSS Statistics 27.0. Categorical variables were presented as frequencies and percentages (%). Normally distributed continuous variables were described as mea*n* ± standard deviation; non-normally distributed continuous variables were described as median with interquartile range [M (P25, P75)]. Collinearity among variables was assessed using the variance inflation factor (VIF), with VIF > 5 indicating significant collinearity. A two-sided P-value < 0.05 was considered statistically significant.

#### 2.5.1 Pre-matching analyses

Between-group comparisons were performed using the *χ*^2^ test for categorical variables, the independent samples t-test for normally distributed continuous variables, and the Mann–Whitney U test for non-normally distributed continuous variables.

#### 2.5.2 Propensity score matching (PSM)

To minimize potential confounding and adjust for baseline differences between the two groups, propensity score matching (PSM) analysis was performed. The covariates included in the PSM were those with significant baseline differences in Table 1, including: gestational age, birth weight, mode of delivery, SGA status, and 5-minute Apgar score ≤7. Using the FOF time grouping as the dependent variable, a 1:1 nearest-neighbor matching algorithm was employed without replacement, with a caliper width of 0.1. After matching, 179 matched pairs (358 infants) were successfully formed for subsequent analyses. Covariate balance after matching was assessed using standardized mean difference (SMD), with an SMD < 0.2 indicating good balance.

**Table 1:** Baseline characteristics before and after propensity score matching between the FOF short group and FOF long group.

Variable		Before PSM (*n* = 753)				After PSM (*N* = 358)		
		FOF Short(*n* = 381)	FOF Long (*n* = 372)	*p-value*	SMD	FOF Short(*n* = 179)	FOF Long(*n* = 179)	SMD
Gestational age, weeks, median (IQR)		30.4 (29.3, 31.8)	28.3 (27.4, 29.6)	＜0.001	1.278	29.6 (28.7, 30.6)	29.3 (28.4, 30.3)	0.225
Birth weight, g, median (IQR)		1,280 (1,150, 1,365)	1,000 (852,1,140)	＜0.001	1.301	1,180 (1,040, 1,280)	1,120 (1,000,1,250)	0.192
Sex, *n* (%)	Female	192 (50.4)	168 (45.2)	0.151	0.105	89 (49.7)	78 (43.6)	0.123
	Male	189 (49.6)	204 (54.8)			90 (50.3)	101 (56.4)	
Mode of delivery, *n* (%)	Vaginal	23 (6.0)	58 (15.6)	＜0.001	0.311	16 (8.9)	19 (10.6)	0.056
	Cesarean section	358 (94.0)	314 (84.4)			163 (91.1)	160 (89.4)	
Plurality, *n* (%)	Singleton	263 (69.0)	237 (63.7)	0.122	0.113	127 (70.9)	110 (61.5)	0.190
	Multiple^a^	118 (31.0)	135 (36.3)			52 (29.1)	69 (38.5)	
SGA, *n* (%)	No	311 (81.6)	332 (89.2)	0.003	0.217	160 (89.4)	164 (91.6)	0.076
	Yes	70 (18.4)	40 (10.8)			19 (10.6)	15 (8.4)	
5-min Apgar score ≤7, *n* (%)	No	323 (84.8)	224 (60.2)	＜0.001	0.572	136 (76.0)	128 (71.5)	0.102
	Yes	58 (15.2)	148 (39.8)			43 (24.0)	51 (28.5)	
Maternal age, years, median (IQR)		32 (28, 35)	32 (29, 36)	0.374	0.104	32 (29, 36)	31 (29, 35)	0.034
Hypertensive disorders of pregnancy, *n* (%)	No	214 (56.2)	230 (61.8)	0.114	0.115	101 (56.4)	103 (57.5)	0.023
	Yes	167 (43.8)	142 (38.2)			78 (43.6)	76 (42.5)	
Gestational diabetes, *n* (%)	No	314 (82.4)	293 (78.8)	0.205	0.092	148 (82.7)	136 (76.0)	0.166
	Yes	67 (17.6)	79 (21.2)			31 (17.3)	43 (24.0)	
Gestational hypothyroidism, *n* (%)	No	359 (94.2)	346 (93.0)	0.495	0.050	171 (95.5)	163 (91.1)	0.179
	Yes	22 (5.8)	26 (7.0)			8 (4.5)	16 (8.9)	
Antenatal corticosteroids, *n* (%)	No	85 (22.3)	80 (21.5)	0.790	0.019	37 (20.7)	34 (19.0)	0.042
	Yes	296 (77.7)	292 (78.5)			142 (79.3)	145 (81.0)	

PSM, propensity score matching; IQR, interquartile range; SMD, standardized mean difference; SGA, small for gestational age; FOF, full oral feeding.

a Multiple refers to twins and higher-order multiples.

#### 2.5.3 Post-matching comparisons

The matched data had a paired structure (179 pairs), and paired methods were used for between-group comparisons. Continuous variables were compared using the Wilcoxon signed-rank test; categorical variables were compared using McNemar's test when applicable, with Fisher's exact test used as an alternative when McNemar's test could not be computed due to zero variance in one group.

### 2.6 Univariate and multivariate logistic regression analysis

Analysis 1 (Overall data): Variables that were statistically significant in the univariate analysis and showed no collinearity were included in a multivariable logistic regression model to identify independent risk factors for prolonged FOF time using the pre-matching dataset.

Analysis 2 (Post-PSM data): Conditional logistic regression was applied to the matched dataset to identify independent risk factors for prolonged FOF time, appropriately accounting for the paired structure of the matched data (179 pairs).

## 3 Results

### 3.1 Baseline characteristics and PSM results

A total of 1,030 VLBWI were initially identified during the study period. After excluding 10 infants with congenital gastrointestinal malformations or genetic metabolic diseases, 82 who died within the first week, 9 with spontaneous intestinal perforation, 14 whose families withdrew treatment for non-medical reasons, and 162 with incomplete clinical data, 753 infants were ultimately included in the analysis. Among them, 393 (52.2%) were male and 360 (47.8%) were female. The median time to achieve FOF for the 753 VLBWIs was 37 (28, 47) days. Based on this median, 372 infants (49.4%) were assigned to the long-FOF group and 381 (50.6%) to the short-FOF group.

### 3.2 Post-matching basic analysis and conditional logistic regression analysis

In the successfully matched cohort (*n* = 358), we further evaluated the differences in mechanical ventilation-related variables between the long- and short-FOF groups (Table 2). After matching, the proportion of infants who received initial invasive mechanical ventilation showed no significant difference between the groups (long vs. short: 31.3% vs. 30.2%, *P* = 0.906). However, the durations of both non-invasive positive pressure ventilation (median: 19 vs. 13 days, *P* < 0.001) and total positive pressure support (median: 23 vs. 15 days, *P* < 0.001) were significantly longer in the long-FOF group. The duration of initial invasive mechanical ventilation showed no statistical difference between the groups (*P* = 0.224).

**Table 2:** Comparison of differences in mechanical ventilation-related variables before and after matching.

Variable		Before PSM			After PSM		
		FOF Short(*n* = 381)	FOF Long(*n* = 372)	*p-value*	FOF Short(*n* = 179)	FOF Long(*n* = 179)	*p-value*
Initial invasive ventilation, *n* (%)	No	309 (81.1)	182 (48.9)	＜0.001	125 (69.8)	123 (68.7)	0.906
	Yes	72 (18.9)	190 (51.1)		54 (30.2)	56 (31.3)	
Non-invasive positive pressure support, *n* (%)	No	18 (4.7)	1 (0.3)	＜0.001	4 (2.2)	0(0)	0.123
	Yes	363 (95.3)	371 (99.7)		175 (97.8)	179 (100)	
Duration of initial invasive ventilation, days, median (IQR)		0 (0, 0)	1 (0, 8)	＜0.001	0 (0, 2)	0 (0, 2)	0.224
Duration of non-invasive positive pressure support, days, median (IQR)		9 (6, 17)	25 (14, 37)	＜0.001	13 (7, 22)	19 (10, 31)	＜0.001
Total duration of positive pressure support, days, median (IQR)		10 (6, 18.5)	32 (18, 46.8)	＜0.001	15 (8, 26)	23 (12, 34)	＜0.001

PSM, propensity score matching; IQR, interquartile range; FOF, full oral feeding.

To analyze the independent association of mechanical ventilation variables with the risk of prolonged time to achieve FOF, logistic regression analyses were performed (Table 3). Univariate analysis showed that the duration of initial invasive ventilation (OR = 1.232, 95% CI: 1.167-1.301), non-invasive positive pressure support (OR = 1.090, 95% CI: 1.075-1.106), and total positive pressure support (OR = 1.087, 95% CI: 1.073-1.101) were all significantly associated with an increased risk of prolonged FOF time.

**Table 3:** Multivariable logistic regression analysis of factors associated with extended time to achieve full oral feeding.

Variable	Unadjusted OR (95%CI)	Model 1:aOR^a^ (95%CI)	Model 2:PS-Matched aOR^b^ (95%CI)	Model 3:aOR^c^ (95%CI)	Model 4:PS-Matched aOR^d^ (95%CI)
Duration of initial invasive ventilation (days)	1.232 (1.167–1.301)	1.065 (1.011–1.123)	1.067 (1.010–1.127)	1.030 (0.976–1.087)	1.036 (0.978–1.096)
Duration of non-invasive positive pressure support (days)	1.090 (1.075–1.106)	1.040 (1.023–1.057)	1.035 (1.015–1.055)	-	-
Total duration of positive pressure support (days)	1.087 (1.073–1.101)	-	-	1.038 (1.021–1.055)	1.033 (1.015–1.052)

a Model 1 (Pre-PSM data): Adjusted for gestational age, birth weight, mode of delivery, and 5-min Apgar score≤7, analyzing the impact of initial invasive ventilation duration and non-invasive support duration.

b Model 2 (Post-PSM data): Analyzing the impact of initial invasive ventilation duration and non-invasive support duration.

c Model 3 (Pre-PSM data): Adjusted for gestational age, birth weight, mode of delivery, and 5-min Apgar score≤7, analyzing the impact of initial invasive ventilation duration and total support duration.

d Model 4 (Post-PSM data): Analyzing the impact of initial invasive ventilation duration and total support duration.

Collinearity diagnosis of variables significant in univariate analysis revealed no collinearity among variables except for the total duration of positive pressure support. In the pre-matching model adjusted for baseline confounders (Model 1), both the duration of initial invasive ventilation (aOR = 1.065, 95% CI: 1.011-1.123) and the duration of non-invasive support (aOR = 1.040, 95% CI: 1.023-1.057) showed statistical significance. When total positive pressure support duration (aOR = 1.038, 95% CI: 1.021-1.055) was analyzed instead of non-invasive support duration in Model 3, the association of initial invasive ventilation duration was no longer significant (aOR = 1.030, 95% CI: 0.976-1.087).

The pattern of results was clearer in the analyses based on the matched sample (Models 2 & 4). The association of initial invasive mechanical ventilation duration was model-dependent: it was significantly associated with prolonged FOF time when adjusted for non-invasive positive pressure support duration (Model 2: aOR = 1.067, 95% CI: 1.010-1.127); however, this association was not statistically significant when adjusted for total positive pressure support duration (Model 4: aOR = 1.036, 95% CI: 0.978-1.096). In contrast, both the duration of non-invasive positive pressure support (Model 2: aOR = 1.035, 95% CI: 1.015-1.055) and the total duration of positive pressure support (Model 4: aOR = 1.033, 95% CI: 1.015-1.052) maintained stable and significant independent associations, indicating an approximately 3.3% increased risk per additional day.

### 3.3 Sensitivity analysis

To assess the robustness of the core findings, we conducted a sensitivity analysis by comparing model results derived from different confounding control strategies (Table 3). First, the associations of non-invasive positive pressure support duration and total positive pressure support duration with prolonged FOF time were highly consistent across both analytical frameworks. Specifically, both were significantly associated with an increased risk of prolonged FOF time in the pre-matching multivariate-adjusted models (non-invasive duration: aOR = 1.040, 95% CI: 1.023-1.057; total duration: aOR = 1.038, 95% CI: 1.021-1.055). In the post-PSM sample, the significant associations for these two variables remained stable, with similar effect sizes (non-invasive duration: aOR = 1.035; total duration: aOR = 1.033). This consistency indicates that longer durations of both non-invasive and total respiratory support are robust and independent risk factors for delayed FOF, and this association is not influenced by the method of baseline confounding control.

The association of initial invasive mechanical ventilation duration varied across models and appeared model-dependent. In the pre-matching adjusted model (Model 1), it was significantly associated with delayed FOF (aOR = 1.065, 95% CI: 1.011-1.123); however, in the PSM-matched sample where baseline differences were eliminated, its association remained significant when adjusted for non-invasive support duration (Model 2: aOR = 1.067, 95% CI: 1.010-1.127), but was not statistically significant when adjusted for total support duration (Model 4: aOR = 1.036, 95% CI: 0.978-1.096). This pattern suggests that the independent effect of initial invasive ventilation duration on FOF delay may be less robust than that of non-invasive support duration, and its association appears to be influenced by model specification.

## 4 Discussion

This study employed propensity score matching to investigate the impact of respiratory support duration on the time to achieve FOF in very low birth weight infants. The main findings indicate that the duration of non-invasive positive pressure support and the total duration of positive pressure support are independent risk factors for prolonging the transition to FOF, and these associations remained robust across different analytical strategies. In contrast, the independent effect of initial invasive mechanical ventilation duration appeared model-dependent.

In the present study, the association between initial invasive mechanical ventilation duration and delayed FOF appeared model-dependent after propensity score matching. This result does not negate its clinical impact but suggests a need for a more nuanced understanding of its pathway of effect. The results of the pre-matching multivariate logistic regression analysis (Model 1) showed that initial invasive mechanical ventilation was an independent risk factor for longer time to achieve FOF; each additional day of initial invasive ventilation duration was associated with a 1.065-fold increased risk of prolonged FOF transition. Regarding the impact of invasive ventilation, the literature presents mixed conclusions. Some studies suggest that invasive mechanical ventilation, due to its greater disruption of systemic physiology, is a strong predictor of feeding delay (13). However, our study did not find a consistent independent effect after matching. This discrepancy may be related to insufficient control for confounding variables such as illness severity in previous studies. The strong association observed before matching likely reflects the fact that infants with more severe illness, lower gestational age, and lower Apgar scores simultaneously required longer invasive ventilation and longer feeding support. After PSM balanced these baseline differences, the “net effect” of invasive ventilation itself diminished.

VLBWI often have immature lung development and are critically ill due to various perinatal risk factors; some require invasive mechanical respiratory support. A longer duration of initial invasive ventilation typically indicates poorer overall function and less mature oral feeding readiness in preterm infants (14), leading to a prolonged time to achieve FOF. Prolonged invasive mechanical ventilation can reduce blood flow distribution to the gastrointestinal tract and weaken gastrointestinal motility (13). Furthermore, endotracheal intubation for invasive ventilation can indeed cause glottic or laryngeal injury. Extremely preterm infants, due to orotracheal intubation and prolonged tube feeding, often experience an open, non-oral state, which reduces stimulation for the suck-swallow-breathe sequence (3). Prolonged mechanical ventilation can lead to ventilator dependence and stimulate pulmonary stretch receptors, potentially inhibiting spontaneous breathing to some extent. This results in poorer coordination of sucking, swallowing, and breathing during feeding, prolonging the transition to oral feeding (15). Tan et al. (16) showed that prolonged use of invasive mechanical ventilation easily leads to ventilator dependence and ventilator-associated pneumonia (VAP), increasing the risk of infection. Similarly, the occurrence of VAP can itself prolong the duration of mechanical ventilation, thereby extending the time to achieve FOF in VLBWI. In addition to providing oral motor interventions to improve infants' sucking and swallowing ability (17), early administration of human milk can be encouraged. Current evidence indicates that human milk not only stimulates the development of swallowing reflexes (18) but also contains various immunologically active factors. Early oropharyngeal administration of colostrum can enhance infant immunity and reduce the incidence of infection (19). Preventing VAP in VLBWI by elevating the head of the bed to 30° to prevent aspiration and gastroesophageal reflux is also an effective measure (20). Furthermore, nursing staff can implement bundled care strategies including strict ward environment management, aseptic techniques, and oral care to reduce infection incidence, prevent VAP, shorten the duration of invasive mechanical ventilation, improve feeding tolerance, and reduce the time to achieve FOF (21).

This study confirmed that longer durations of non-invasive ventilation and total positive pressure respiratory support were significantly associated with delayed FOF. Each additional day of non-invasive positive pressure support increased the risk of delayed FOF by approximately 3.5%. This finding aligns with several recent studies. A meta-analysis noted that the duration of positive pressure ventilation is a major obstacle to the progression of oral feeding in preterm infants (11). Similarly, our previous work found that respiratory support delayed the feeding progression in VLBWI and was associated with extrauterine growth retardation (22). Brun et al. (9), in an analysis dividing a cohort of very preterm infants into two subgroups, concluded that the duration of non-invasive ventilation and oxygen therapy was significantly associated with a later postmenstrual age at the achievement of full oral feeding. Similarly, Er et al. (23) indicated that a non-invasive ventilation duration of ≤3 days facilitated an earlier transition to full oral feeding in preterm infants, consistent with the results of this analysis. By employing PSM to further control for key confounders such as gestational age and birth weight, this study strengthens the causal plausibility of the inference that “prolonged respiratory support duration directly affects feeding function.”

In most NICUs, healthcare providers may withhold oral feeding for infants on respiratory support due to fear of aspiration, thereby prolonging the time to achieve FOF (9). On the other hand, the noxious stimuli from the respiratory support therapy itself can also affect oral feeding ability (4). Non-invasive positive pressure support, while meeting the oxygenation needs of VLBWI, often leads to increased air swallowing due to the continuous positive airway pressure. This increases gastrointestinal gas and slows motility, causing decreased pre- and postprandial intestinal blood flow (24). Additionally, repeated oropharyngeal stimulation from the presence of a feeding tube during nasal CPAP support can contribute to gastrointestinal dysfunction, commonly manifesting as abdominal distension and vomiting (25), thereby delaying oral feeding progression (24). Furthermore, the continuous positive airflow may disrupt the fragile integration of the suck-swallow-breathe rhythm in preterm infants. To counteract or adapt to this non-physiological airflow, infants may exhibit delayed or ineffective swallowing, prolonging the oral feeding process (26).

Based on the above mechanisms, during the administration of non-invasive ventilation, clinicians can actively employ a series of non-pharmacological interventions to mitigate its negative effects. For example, implementing prone positioning when the infant's condition allows. Jin et al. (27) found that in the prone position, infants showed increased ventilation in the lower lung fields and improved arterial oxygenation, which is beneficial for gastric emptying. Simultaneously, compression of the abdomen can promote intestinal peristalsis, effectively alleviating abdominal distension during non-invasive positive pressure ventilation and reducing the incidence of gastrointestinal complications. Abdominal massage and cleansing enemas are also important measures to relieve abdominal distension and vomiting, effectively promote gastrointestinal motility, significantly reduce the occurrence of gastrointestinal adverse reactions, and shorten the time to achieve FOF (28). The neonatal pulmonary rehabilitation training protocol proposed by Song et al. (29) can also shorten the time to achieve FOF. This protocol includes interventions such as passive upper limb exercise training, resistance training, and inspiratory muscle training, which improve respiratory function and circulation, thereby shortening the duration of non-invasive positive pressure ventilation. Additionally, encouraging Kangaroo Mother Care (KMC) may help reduce the duration of non-invasive positive pressure support. A prospective study by Bisanalli et al. demonstrated that KMC is feasible and safe for low birth weight infants on CPAP or SIMV, with no adverse events reported, and is beneficial for low birth weight infants (30). This aligns with the findings of a retrospective cohort study by Xie et al. (31), where the duration of NIPPV and CPAP was significantly shorter in the extremely low birth weight infant group that received KMC compared to the group that did not. Initiating oral feeding early can, to some extent, promote the development of sucking and swallowing functions in preterm infants, thereby facilitating earlier achievement of FOF (4). Oral feeding can be attempted for preterm infants on non-invasive respiratory support, although it is considered a somewhat risky feeding strategy (32). Currently, several studies have shown that oral feeding during non-invasive positive pressure ventilation is safe (33) and may accelerate the progression to oral feeding (34). For example, Dalgleish et al. (33) employed a progressive infant-driven strategy in their study: initially cultivating oral skills via non-nutritive sucking, then starting with 5% of the total daily enteral feeding volume for 5 min per session, gradually increasing the oral feeding amount and time. Respiratory support was maintained during feeding, with real-time monitoring of heart rate and oxygen saturation; feeding was stopped immediately if signs of distress appeared. Additionally, using slow-flow nipples or direct breastfeeding was prioritized to adapt to the infant's sucking rhythm and reduce risk. However, some studies have pointed out that adverse reactions, including apnea and oxygen desaturation, may occur during oral feeding with non-invasive support (35).

## 5 Strengths and limitations

The strength of our study lies in being the first to systematically apply the propensity score matching method in investigating the relationship between respiratory support and feeding outcomes in VLBWI. Through matching, we effectively balanced significant baseline differences in key characteristics such as gestational age, birth weight, and mode of delivery between the long- and short-FOF groups. This greatly reduced confounding bias and strengthened the causal inference regarding the association between prolonged respiratory support duration and feeding delay. Secondly, unlike previous studies that often treated respiratory support as a single variable, this study distinguished and simultaneously analyzed the independent and joint effects of the duration of initial invasive mechanical ventilation, non-invasive positive pressure support, and total positive pressure support. This detailed approach aids in more precisely identifying key targets for clinical intervention. Third, the study was based on a large single-center cohort, with all data derived from a standardized clinical database, ensuring consistency in variable definitions. Focusing on the high-risk population of very low birth weight infants enhances the clinical relevance of the findings for this specific group.

However, this study also has limitations. First, as a single-center retrospective study, the data originated from a single tertiary hospital, whose specific respiratory management strategies, feeding advancement protocols, and nursing practices may be center-specific, thus caution is needed when generalizing the conclusions. Retrospective studies are also inevitably subject to data recording bias. Second, due to the retrospective design, this study lacked detailed data on key variables specifically related to oral feeding readiness, such as neurobehavioral organization, non-nutritive sucking ability, and suck-swallow-breathe coordination. These factors are well-documented determinants of feeding progression in preterm infants (36–38). Therefore, the observed associations between durations of respiratory support and delayed full oral feeding should be interpreted as statistical correlations rather than independent causal effects. Future studies employing prospective multicenter designs are warranted to further validate these associations and to explore the potential interaction mechanisms between respiratory support and feeding outcomes.

## 6 Conclusions

This study suggests that among very low birth weight infants, the duration of non-invasive positive pressure respiratory support is significantly associated with prolonging the transition to full oral feeding. Its effect may be mediated through multiple pathways, including disruption of suck-swallow-breathe coordination and induction of intestinal gas accumulation. Clinically, strategies aiming to shorten the duration of non-invasive support and implementing proactive feeding rehabilitation during this period should be considered core components of optimizing feeding outcomes. Future research should adopt multicenter prospective designs, incorporating detailed recording of nursing interventions, to further validate these findings and explore the underlying specific physiological mechanisms in greater depth.

## Data Availability

The original contributions presented in the study are included in the article/Supplementary Material, further inquiries can be directed to the corresponding authors.
